# *Wolbachia* modify host cell metabolite profiles in response to short‐term temperature stress

**DOI:** 10.1111/1758-2229.70013

**Published:** 2024-09-23

**Authors:** Yu‐Xi Zhu, Yi‐Yin Zhang, Xin‐Yu Wang, Yue Yin, Yu‐Zhou Du

**Affiliations:** ^1^ Department of Entomology, College of Plant Protection Yangzhou University Yangzhou Jiangsu China; ^2^ Department of Entomology, College of Plant Protection Nanjing Agricultural University Nanjing Jiangsu China; ^3^ Institute for the Control of the Agrochemicals Ministry of Agriculture and Rural Affairs Beijing China

## Abstract

*Wolbachia* are common heritable endosymbionts that influence many aspects of ecology and evolution in various insects, yet *Wolbachia*‐mediated intracellular metabolic responses to temperature stress have been largely overlooked. Here, we introduced the *Wolbachia* strain wLhui from the invasive *Liriomyza huidobrensis* (Blanchard) into a *Drosophila* Schneider 2 cell line (S2) and investigated the metabolite profile of wLhui‐infected (S2_wLhui) and uninfected cell lines (S2_wu) under short‐term exposure to either high (37°C), moderate (27°C), or low (7 and 17°C) temperatures. We find that *Wolbachia* infection, temperature stress, and their interactions significantly affect cellular metabolic profiles. Most significantly, when comparing the changes in metabolites between S2_wLhui and S2_wu, glycerophospholipids, amino acids, and fatty acids associated with metabolic pathways, microbial metabolism in diverse environments, and other pathways were significantly accumulated at either low or high temperatures. Our findings suggest *Wolbachia*‐induced cellular physiological responses to short‐term temperature stress, which may in turn affect the fitness and adaptive ability of its host as an invasive species.

## Graphical abstract

The wLhui strain from the leaf miner successfully colonized the *Drosophila* Schneider 2 cell line. We discovered that *Wolbachia* induced substantial shifts in the cellular metabolome profile in a temperature‐dependent manner. These metabolite profile modifications have significant fitness consequences, which are crucial for understanding the evolution of host–symbiont interactions in the context of environmental temperature changes.

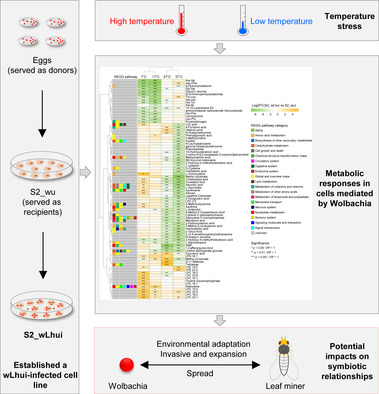

## INTRODUCTION

*Wolbachia* are heritable endosymbionts commonly found in arthropods and known to serve various functions for the host (Werren et al., 2008; Adams et al., 2021). Most *Wolbachia* strains form a stable symbiotic relationship with their insect hosts (Porter & Sullivan, 2023), but such symbioses are often challenged by extreme temperatures (Corbin et al., 2017). Both high and low temperatures lead to the loss of *Wolbachia* in various insect species, which causes the breakdown of the symbiotic relationship and thus also influences aspects of host physiology, behaviour, and overall fitness affected by the symbiotic association (Hague et al., 2022; Ross et al., 2019). To maintain the symbiosis, *Wolbachia* might aid the host in adapting to or escaping temperature stress through behavioural, physiological, and molecular responses, such as altering thermoregulatory behaviour (Truitt et al., 2019; Hague et al., 2020; Strunov et al., 2024), regulating host transcription responses (Zhu et al., 2021) or changing nutritional metabolism (Hosokawa et al., 2010; Ju et al., 2020). Elucidating how *Wolbachia*–host symbioses respond to temperature stress can provide insights into the stability and adaptive capability of such associations, with profound implications for understanding the interaction between endosymbionts and hosts in the face of environmental change.

Several recent surveys used direct comparisons of *Wolbachia*‐infected and uninfected hosts to demonstrate that *Wolbachia* infections can strongly affect insect host metabolism (Karpova et al., 2023; Kryukova et al., 2023; Li et al., 2022; Zhang et al., 2021). However, *Wolbachia* generally alters the microbiome of infected insects (Duan et al., 2020), and thus these comparisons might not rule out the synergistic influence of *Wolbachia* and other microbes on host metabolism. Monocausal effects of *Wolbachia* on host metabolism are seldom identified in this manner. In addition, challenges to manipulating *Wolbachia* arise from its obligate intracellular lifestyle and inability to divide outside a host cell, hampering the elucidation of their potential functions for the host (Kaur et al., 2021). Fortunately, these limitations can be overcome by analysis of cell lines with artificial transinfection of special *Wolbachia* strains and metabolomics (Hayward & Colinet, 2023; Li et al., 2021; Molloy et al., 2016). Prior studies applying this methodology found that *Wolbachia* modulate lipid and other metabolites in cells of the mosquito *Aedes albopictus*, and thus might block the transmission of viruses or mediate host response to temperature stress (Li et al., 2021; Molloy et al., 2016). Similar analyses could be utilized to better understand the effect of *Wolbachia* on metabolisms in a broader range of insect hosts in terms of temperature stress, an area where few data have been published.

Invasive insect species seriously threaten agricultural and natural ecosystems worldwide (Diagne et al., 2021), and heritable symbionts within invasive insect hosts may facilitate or restrain host adaption and expansion in a variety of ways (Lemoine et al., 2020; Lu et al., 2016). The invasive leaf miner *Liriomyza huidobrensis*, first detected in Yunnan, China in 1993, has rapidly spread to multiple cool regions of China (Chen & Kang, 2004). Our previous study suggested that this notorious pest species is frequently infected with *Wolbachia* (Zhu et al., 2022). Here, we further elucidate the implications of this symbiotic relationship by examining how *Wolbachia* could manipulate host metabolites to face temperature changes during invasions. We first newly introduce the *Wolbachia* strain wLhui into a *Drosophila* Schneider 2 cell line (S2) to stably establish a wLhui‐infected cell line. Then, we compared the metabolic profile between wLhui‐infected and ‐uninfected cell lines combined with short‐term exposure to high or low temperatures using nontargeted metabolomics.

## EXPERIMENTAL PROCEDURES

### Wolbachia *strain and cell line*

The *Wolbachia* strain wLhui was extracted from the invasive leaf miner *L. huidobrensis*. The *L. huidobrensis* specimens were originally collected from Yuanmou Country, Yunnan Province, China in 2023. Wild‐type leaf miner populations were found to be naturally infected at a 100% prevalence with the *Wolbachia* strain wLhui, and wLhui could be stably passaged under laboratory conditions at 25 ± 1°C, 60% relative humidity and under 16 h light: 8 h dark lighting cycle. The *Wolbachia* wLhui strain was identified using multi‐locus sequence typing (MLST) (https://pubmlst.org/organisms/wolbachia-spp/) as described previously (Xia et al., 2018). Phylogenetic analyses were performed using Bayesian Inference and maximum‐likelihood estimation for a concatenated data set of MLST genes by MEGA11 (Xia et al., 2018).

The *Drosophila* Schneider 2 cell line (S2) was not infected by *Wolbachia* (S2_wu) and was kindly provided by Professor Xiao‐Yue Hong of the Department of Entomology, College of Plant Protection, Nanjing Agriculture University.

### *Transinfection of* Wolbachia *into the S2 cell line*

We introduced the *Wolbachia* wLhui into the Schneider 2 cell line S2_wu to establish a stable wLhui‐infected cell line (S2_wLhui) following previously described methods (Li et al., 2022). In brief, mature fertilized females of leaf miners were placed in a clip cage containing bean seedlings and allowed to lay eggs for 12 h. The newly laid eggs were picked with a sterile needle and immersed in 1× phosphate‐buffered saline (PBS). To eliminate potential microbes on the surface of eggs, these eggs were washed with PBS for 5 min, 2% liquor natrii hypochloritis for 1.5 min, 75% ethanol for 3 min, and PBS again for 5 min. The ~4000 sterilized eggs were immersed and ground in culture mediums. The grinding solutions used were poured into culture mediums with S2_wu cells in 6‐well plates. The plates were centrifuged at 350× *g* for 3 h at 4°C and then cultured in Schneider's *Drosophila* medium with 20% foetal bovine serum at 27°C in a dark incubator. After 5 days, the transinfected cells were transferred into new cell culture flasks and then passaged to new culture flasks every 7 days thereafter (Figure S1). DNA was extracted from the S2_wLhui cells and tested for *Wolbachia* infection status by PCR as described previously (Li et al., 2022).

### Temperature treatment on cell lines

To determine the effect of *Wolbachia* on cell host metabolic responses to short‐term high or low temperatures, the cell lines with and without *Wolbachia* wLhui at 21 generations were exposed to temperatures of 7, 17, 27, or 37°C for 6 h. A total of 1 × 10^7^ cells from each sample were collected and frozen in liquid nitrogen for 15 min. Six replicates for each treatment were used to extract metabolites (Figure S1).

### Metabolomic determination

Metabolites of 48 cell samples from *Wolbachia*‐infected or ‐uninfected cell lines given different temperature treatments were investigated using non‐targeted metabolomics. Metabolites were extracted from each sample using prechilled 80% methanol. The mixture samples were incubated on ice for 5 min and then were centrifuged at 15,000 rpm, 4°C for 5 min. The supernatant was collected and then metabolites were analysed using ultra‐high‐performance liquid chromatography–tandem mass spectrometry (UHPLC–MS/MS). The data from positive‐ion and negative‐ion modes were collected separately to improve the coverage of metabolites. UHPLC–MS/MS analyses were performed using a Vanquish UHPLC system (Thermo Fisher, Germany) coupled with an Orbitrap Q Exactive™ HF mass spectrometer (Thermo Fisher, Germany) in Biozeron Co., Ltd (Shanghai, China). The raw data files generated by UHPLC–MS/MS were processed using Compound Discoverer 3.1 (Thermo Fisher) to perform peak alignment, peak picking, and quantification for each metabolite. Metabolites were annotated using the KEGG database (https://www.genome.jp/kegg/pathway.html), HMDBdatabase (https://hmdb.ca/ metabolites) and LIPID Maps database (http://www.lipidmaps.org/) as references.

### Statistical analysis

All statistical analyses were performed and visualized using either R (version 3.3.2), MetaboAnalyst 6.0 (https://www.metaboanalyst.ca/), or OmicShare platform tools (https://www.omicshare.com/tools). Principal component analysis (PCA) and partial least squares discriminant analysis were applied to screen and compare the metabolites among different cell line samples. A permutational multivariate analysis of variance (PERMANOVA) was then conducted to investigate the significant effect of *Wolbachia* infection status, temperature treatments, and their interactions on cell line metabolites (Bartel et al., 2013; Pérez‐Cova et al., 2022). Further, a Procrustes analysis was used to test the correlation between metabolites and temperature or *Wolbachia* without considering the interaction effects of both factors on cell metabolism. The differential metabolites between S2_wLhui and S2_wu cells under each temperature treatment were screened using to the following parameters: log2 (Fold Change) and ‐log10 (*p*‐value) of metabolites. The metabolites with variable importance in projection (VIP) scores >1 and *p*‐value <0.05 and FC ≥ 2 or FC ≤0.5 were considered to be differential metabolites. The functions of these metabolites and metabolic pathways were studied using the KEGG database. Analysis of metabolic pathway enrichment of differential metabolites was performed, and metabolic pathways with a *p*‐value <0.05 were considered statistically significantly enriched.

## RESULTS

**FIGURE 1:**
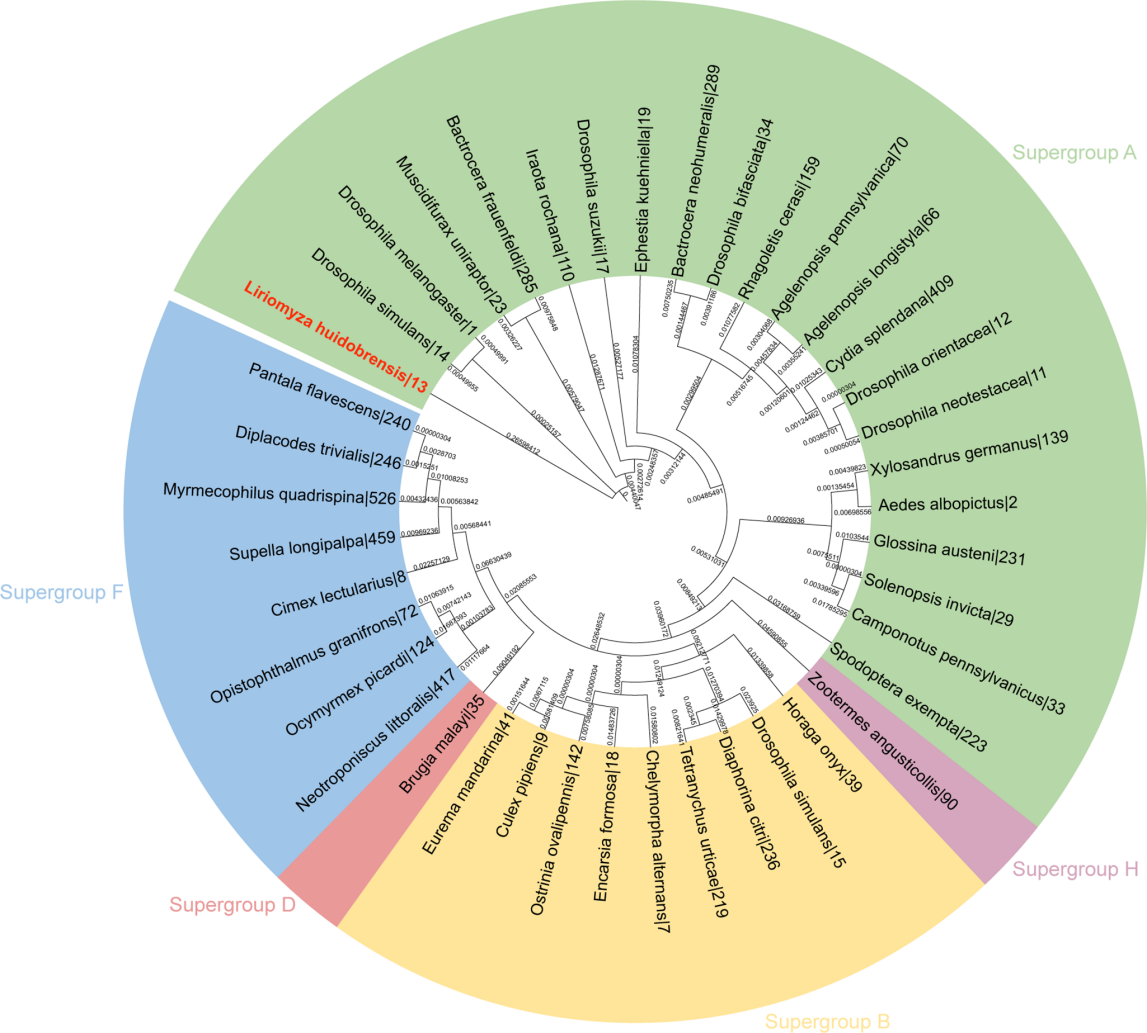
Phylogenetic analysis of the leaf miner *Wolbachia* strain wLhui was conducted using concatenated multi‐locus sequence typing (MLST) data. The numbers on the branches represent length measured in the number of substitutions per site. wLhui is highlighted in red, and other *Wolbachia* strains represent supergroups A, B, D, F, and H. Strains are characterized by the names of their host species and ST number from the MLST database.

Phylogenetic analysis based on the MLST sequence showed that the wLhui strain belonged to supergroup A (Figure 1). The *Wolbachia* wLhui was successfully transinfected into the *Drosophila* Schneider 2 cell line (S2) and stably transmitted during cell passage for more than 20 generations (Figure S2).

A total of 1162 metabolites were obtained after quality control, with 669 and 493 metabolites respectively detected in the positive‐ion model and negative‐ion model (Table S1). A total of 29.86%, 46.99%, and 14.97% of metabolites were annotated in KEGG (347/1162), HMDB (546/1162), and Lipidmaps (174/1162), respectively. Overall, most of the metabolites were organic acids and their derivatives (12.91%), followed by lipids and lipid‐like molecules (8.86%), and benzenoids (8.43%) (Figure S3).

**FIGURE 2:**
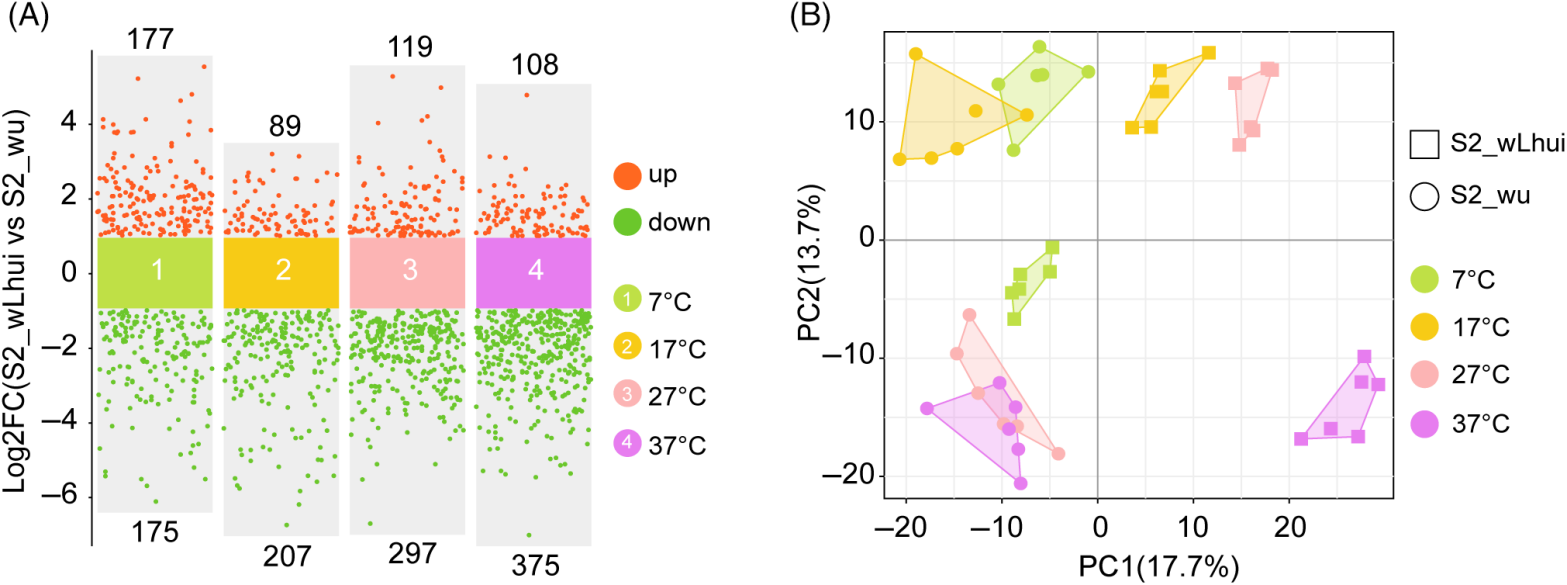
(A) The number of differential metabolites between wLhui‐infected and uninfected cell lines exposed to each temperature. (B) Principal component (PC) analysis shows the clustering of samples from different treatments. The coordinate axis represents the percentage of contribution of each PC to the total variance.

**FIGURE 3:**
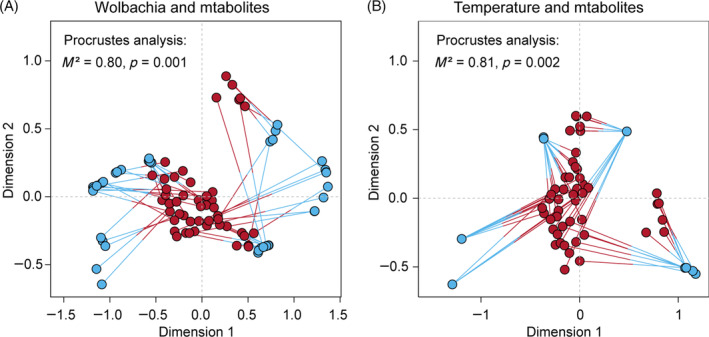
Procrustes analysis of the correlation between metabolites and *Wolbachia* infection status (A) or temperature treatments (B). Red dots represent the metabolomes, while blue dots represent *Wolbachia* infection status or temperatures. The significance level: *p* < 0.05.

The total number of significantly differential metabolites between S2_wLhui and S2_wu was highest at 37°C (*n* = 483, of which 108 were upregulated and 375 downregulated), followed by 27°C (*n* = 416, 119 upregulated and 297 downregulated), then 7°C (*n* = 352, 177 upregulated, 175 downregulated), and the lowest was at 17°C (*n* = 296, 89 upregulated and 207 downregulated; Figures 2A and S4 and Table S2). PCA captured 31.4% of the variance in metabolites of the two cell lines under different temperatures, with PC1 = 17.7% and PC2 = 13.7% (Figure 2B). The PERMANOVA analysis revealed significant effects of *Wolbachia* infection status (*R* ^2^ = 0.17, *p* < 0.001), temperature treatment (*R* ^2^ = 0.31, *p* < 0.001), as well as their interaction (*R* ^2^ = 0.35, *p* < 0.001), in explaining the variance of metabolites. The Procrustes analysis, in which interactions were not considered, further indicated that the metabolite concentration of the S2 cell line was significantly correlated with both the *Wolbachia* infection status (*M* ^2^ = 0.80, *p* = 0.001) and the temperature treatment (*M* ^2^ = 0.81, *p* = 0.002; Figure 3). The top 25 metabolites correlated with temperature or *Wolbachia* infection are shown in Figure S5. Among them, choline and *N*‐acetylgluconsamine 1‐phosphate were positively correlated with *Wolbachia* infection, while some Glycerophospholipids were positively correlated with temperature (Figure S5).

**FIGURE 4:**
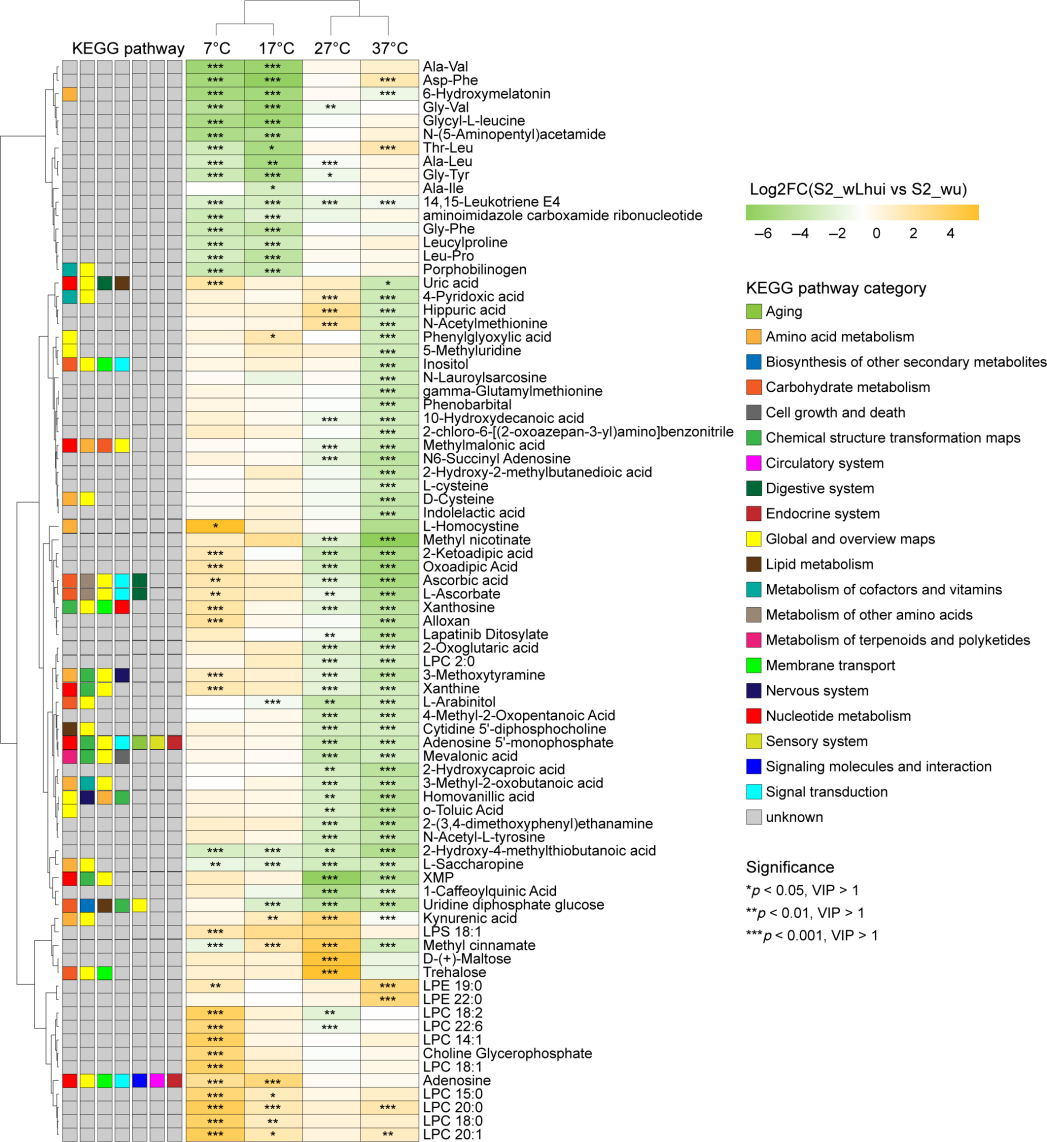
The significantly differential metabolites and associated KEGG pathways between wLhui‐infected and uninfected cell lines at each temperature. The significance level: *, *p* < 0.05, VIP >1; **, *p* < 0.01, VIP >1; ***, *p* < 0.001, VIP >1. VIP, variable importance in projection.

The differential metabolites between S2_wLhui and S2_wu varied by temperature treatment: trehalose, D‐(+)‐Maltose, methyl cinnamate, LPS 18:1, and Kynurenic acid accumulated under the control temperature (27°C), LPE 22:0 and LPE 19:0 accumulated at 37°C (log_2_[S2_wLhui vs. S2_wu] >3, *p* < 0.001, VIP >1), while XMP, 1‐Caffeoylquinic Acid, Uridine diphosphate glucose, L‐arabinitol, 2‐ketoadipic acid, mevalonic acid, adenosine 5′‐monophosphate, 4‐methyl‐2‐oxopentanoic acid, 2‐hydroxy‐4‐methylthiobutanoic acid, oxoadipic acid, L‐saccharopine, 2‐hydroxycaproic acid, 2‐(3,4‐dimethoxyphenyl) ethanamine, cytidine 5′‐diphosphocholine, *N*‐acetyl‐L‐tyrosine and L‐Saccharopine were depleted at control (27°C) and high temperatures (37°C) (log_2_(S2_wLhui vs. S2_wu) >3, *p* < 0.001, VIP >1); L‐Homocystine, LPC 20:1, LPC 20:0, LPC 18:2, LPC 14:1, LPC 18:0, LPC 18:1, LPC 15:0, choline glycerophosphate and LPC 22:6 significantly increased at 7°C; adenosine concentration significantly increased at 17°C, yet Leu‐Pro, Thr‐Leu, leucylproline, Gly‐Tyr, Gly‐Phe, porphobilinogen, Gly‐Val, *N*‐(5‐aminopentyl)acetamide, glycyl‐L‐leucine, 6‐hydroxymelatonin, Asp‐Phe, Ala‐Val were reduced at 7°C or 17°C (log_2_(S2_wLhui vs S2_wu) >3, *p* < 0.001, VIP >1; Figure 4). KEGG enrichment analyses showed that upregulated or downregulated metabolites were mainly related to metabolic pathways, microbial metabolism in diverse environments, carbon metabolism, fatty acid metabolism, biosynthesis and other pathways, and were associated with temperature treatments (Figures 4 and S6 and Table S3).

## DISCUSSION

We observed a distinct disparity between metabolite profiles of wLhui‐infected and uninfected cells. The metabolites that differed between infected and uninfected cell lines also varied under low, moderate, and high‐temperature stresses. Certain metabolites play crucial roles in the physiological properties of insect hosts (Boardman, 2024; Perez & Aron, 2023). Thus, these modifications could have important implications for maintaining symbioses, *Wolbachia* spread, and host fitness.

It is recognized that the success of artificial transinfection strongly depends on the phylogenetic relationship between the donor and the receptor, the *Wolbachia* strain being transinfected, and the status of the recipient (Xia et al., 2018). The success rate of artificial transinfection of *Wolbachia* between closely related hosts is usually higher than distant hosts, which may be related to host immunity (Xia et al., 2018). Our results showed that the wLhui strain from the leaf miner successfully colonized into the *Drosophila* Schneider 2 cell line, suggesting *Wolbachia* can be transinfected between distantly related hosts. Similar results were previously observed using spider mites and the brown planthopper, where the *w*Ttru, *w*Fur, and *w*Lug strains were also successfully transinfected into the *Ae. albopictus* cell line Aa23 (Li et al., 2021; Xia et al., 2018). Our work further demonstrates that the use of cell lines can enable new biochemical investigations into *Wolbachia*‐induced metabolite manipulations to facilitate investigations into responses to temperature stress in various insect species.

Our findings support the view that the cellular metabolome often undergoes major changes in the presence of *Wolbachia* at suitable temperatures. Recent studies on *Habrobracon hebetor* and *Drosophila melanogaster* have shown that *Wolbachia* modifies the metabolism of lipids and carbohydrates in its hosts (Karpova et al., 2023; Kryukova et al., 2023), which may indirectly contribute to host cold tolerance (Boardman, 2024; Perez & Aron, 2023). Here, Trehalose and D‐(+)‐Maltose were significantly stored in wLhui‐infected cells, although the roles of these metabolites in regulating host physiological properties and ability to spread geographically remain to be tested directly in leaf miners. Also, we found that choline was strongly correlated with *Wolbachia* infections, suggesting that *Wolbachia* might supplement choline to host cells. Consistent with our findings, work on other insects has shown that *Wolbachia* supplements biotin and riboflavin to the host, possibly influencing host performance (Ju et al., 2020; Moriyama et al., 2015; Ren et al., 2020; Serrato‐Salas & Gendrin, 2023). Previous studies have suggested that *Wolbachia* affect the synthesis and metabolism of carbohydrates, lipids, amino acids, vitamins, and cofactors in cell lines; the discrepancies across research systems may stem from variances in the host or *Wolbachia* species (Li et al., 2021; Molloy et al., 2016). The specific mechanisms of these modulations have yet to be elucidated. One possibility is that *Wolbachia* directly synthesizes the specific metabolites with its own metabolic enzymes. However, analysis of *Wolbachia* genomes from various insect hosts shows that they are relatively simple and lack the relevant nutrient synthesis‐related genes and that *Wolbachia* even acquires some nutrients from the host (Pramono et al., 2024; Renoz et al., 2019). Future work should combine analysis of the wLhui genome with the metabolome of infected and uninfected hosts to explore this possibility. Another possibility is that the changes in metabolites involve a host response to *Wolbachia* infection or the manipulation of host pathways by *Wolbachia* effectors (Russell et al., 2013). It is not clear which of these hypotheses might apply to our results.

Another notable finding is that *Wolbachia* appears to mediate cellular metabolites in multiple ways in the face of low and high‐temperature stress. This means that changes in cell metabolites are likely linked to trade‐offs within the symbiosis under temperature stresses. Interestingly, while theory suggests that low temperatures may decrease metabolic enzyme activity (Denlinger & Lee Jr, 2010), we detected various *Wolbachia*‐induced metabolites, including many lipids, under low temperatures. In contrast, many metabolites were depleted at high temperatures. The metabolites in which changes were observed were mainly related to metabolic pathways, microbial metabolism in diverse environments, carbon metabolism, fatty acid metabolism, biosynthesis, and other pathways. This implies that *Wolbachia* may produce a cellular‐specific environment to compensate the host in response to low‐temperature environmental conditions or antagonistic to high‐temperature stresses to maintain the symbiotic relationship. The specific metabolites in deferential systems might arise partly through host functional requirements and specific selection across host–microbiota coevolution (Gruntenko et al., 2017; Renoz et al., 2019). The findings presented provide new directions for research into *Wolbachia*‐induced modulation of native host leaf miner metabolism and its ecological consequences.

It is also worth noting that the present study focused on the impact of short‐term temperature treatments on the cell lines. Considering the unstable symbioses between insects and *Wolbachia* under long‐term temperature stress (Hague et al., 2022; Zhu et al., 2021), long‐term temperature treatments are required to further evaluate the evolutionary responses of mutualistic leaf miner–*Wolbachia* symbioses in a natural world of fluctuating temperatures. The association between *Wolbachia* titre and metabolic differences under different temperatures should also be investigated. In addition, metabolomics may be confounded by the cell medium, and the cell lines used in this study were from *Drosophila* instead of the native host of the investigated *Wolbachia* strain. Thus, cell metabolites do not represent native host metabolites (Molloy et al., 2016), and functional assessments of metabolite contribution to phenotype are often lacking. An important next step will be exploring the causes and consequences of *Wolbachia*‐induced native host metabolites. Nevertheless, our results shed insight into *Wolbachia*–host cellular interactions, which potentially lead to promising applications in controlling insect pests (Gong et al., 2023).

## CONCLUSIONS

In summary, our work demonstrates an effective approach using cell lines to investigate *Wolbachia*‐mediated cellular metabolite modification. We found that *Wolbachia* induced substantial shifts in the cellular metabolome profile in a temperature‐dependent manner. These metabolite profile modifications have significant fitness consequences, which are crucial for understanding the evolution of host‐symbiont interactions in the context of environmental temperature changes.

## AUTHOR CONTRIBUTIONS

**Yu‐Xi Zhu:** Conceptualization; software; data curation; writing—original draft; writing—review and editing; funding acquisition; visualization; project administration. **Yi‐Yin Zhang:** Methodology; investigation; software; formal analysis. **Xin‐Yu Wang:** Investigation; methodology. **Yue Yin:** Methodology; investigation; writing—original draft. **Yu‐Zhou Du:** Writing—review and editing; conceptualization; validation; supervision; resources.

## CONFLICT OF INTEREST STATEMENT

The authors declare no conflicts of interest.

## Supporting information


**TABLE S1.** The metabolome dataset shows metabolite information of each cell line.
**TABLE S2.** The significantly differential metabolites.
**TABLE S3.** The metabolic pathway enrichment of differential metabolites.

**FIGURE S1.** Schematic diagram of established *Wolbachia* wLhui‐infected cell lines.
**FIGURE S2.** PCR detection of *Wolbachia* in wLhui‐infected (S2_wLhui) and uninfected (S2_wu) cell lines.
**FIGURE S3.** Classification of indented metabolites.
**FIGURE S4.** Venn diagram of differential metabolites.
**FIGURE S5.** Top 25 metadata correlated with the temperature treatments (A) or the *Wolbachia* infection status (B).
**FIGURE S6.** KEGG enrichment analysis of difference metabolites.

## Data Availability

The data that supports the findings of this study are available in the Appendix S1 and Supplementary Material S1 of this article. All raw metabolome data are available in Table S1. R code used for statistical analyses related to this paper is available on GitHub: https://github.com/yuxizhu0404/Zhu2024.
