# Supplementary material for: Wolbachia modify host cell metabolite profiles in response to short‐term temperature stress
Source: Environ Microbiol Rep. 2024 Sep 23;16(5):e70013. doi: 10.1111/1758-2229.70013 (PMC11420292; doi:10.1111/1758-2229.70013)
Supplement: Supplementary file 2 — FIGURE S1. Schematic diagram of established Wolbachia wLhui‐infected cell lines. FIGURE S2. PCR detection of Wolbachia in wLhui‐infected (S2_wLhui) and uninfected (S2_wu) cell lines. FIGURE S3. Classification of indented metabolites. FIGURE S4. Venn diagram of differential metabolites. FIGURE S5. Top 25 metadata correlated with the temperature treatments (A) or the Wolbachia infection status (B). FIGURE S6. KEGG enrichment analysis of difference metabolites. [file EMI4-16-e70013-s001.docx]

APPENDIX

APPENDIX FIGURES


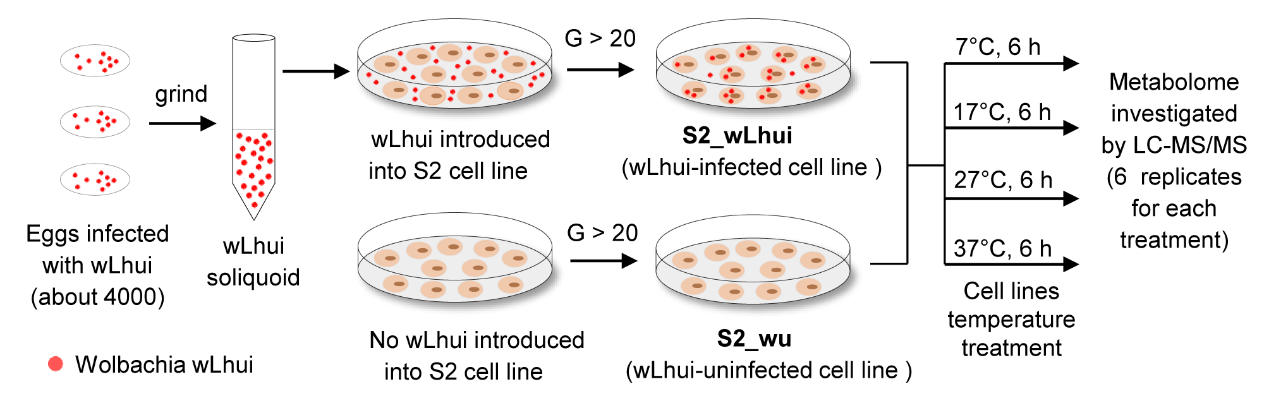


FIGURE S1 Schematic diagram of established *Wolbachia* wLhui-infected cell lines.


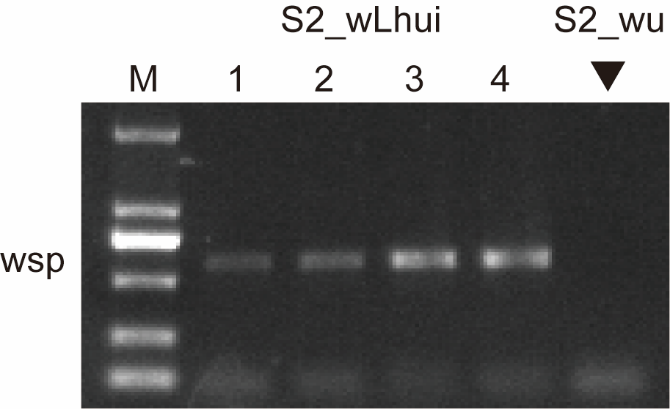


FIGURE S2 PCR detection of *Wolbachia* in wLhui-infected (S2_wLhui) and uninfected (S2_wu) cell lines.


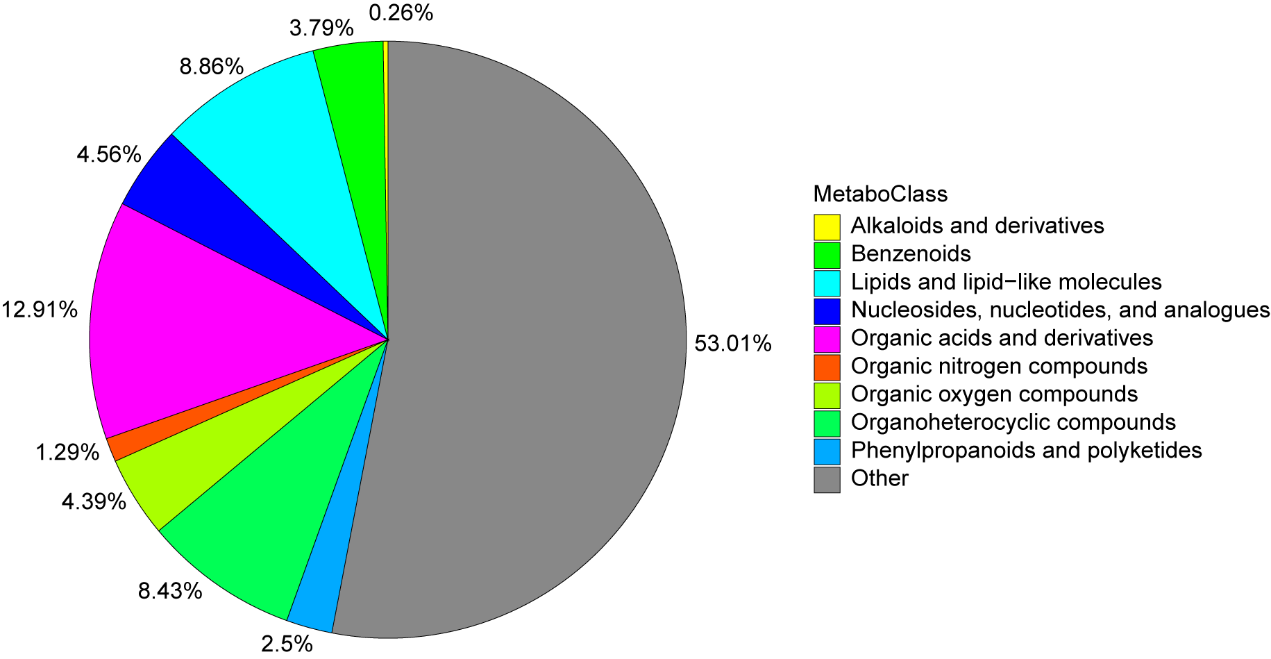


FIGURE S3 Classification of indented metabolites.


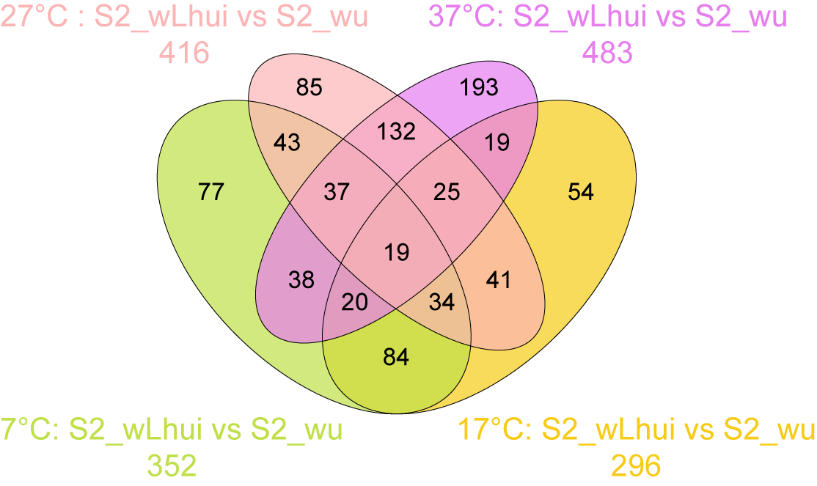


FIGURE S4 Venn diagram of differential metabolites.


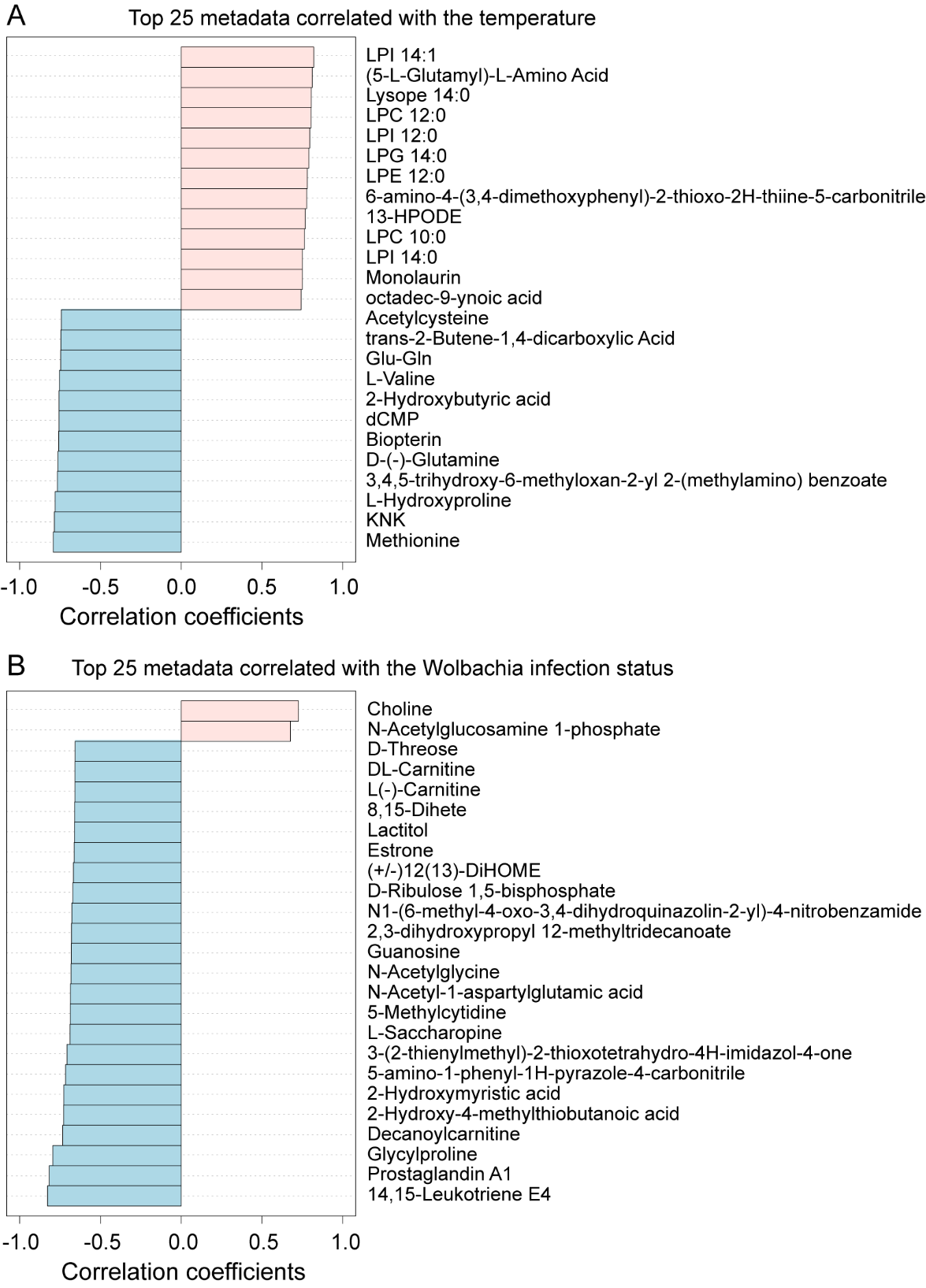


FIGURE S5 Top 25 metadata correlated with the temperature treatments (A) or the *Wolbachia* infection status (B).


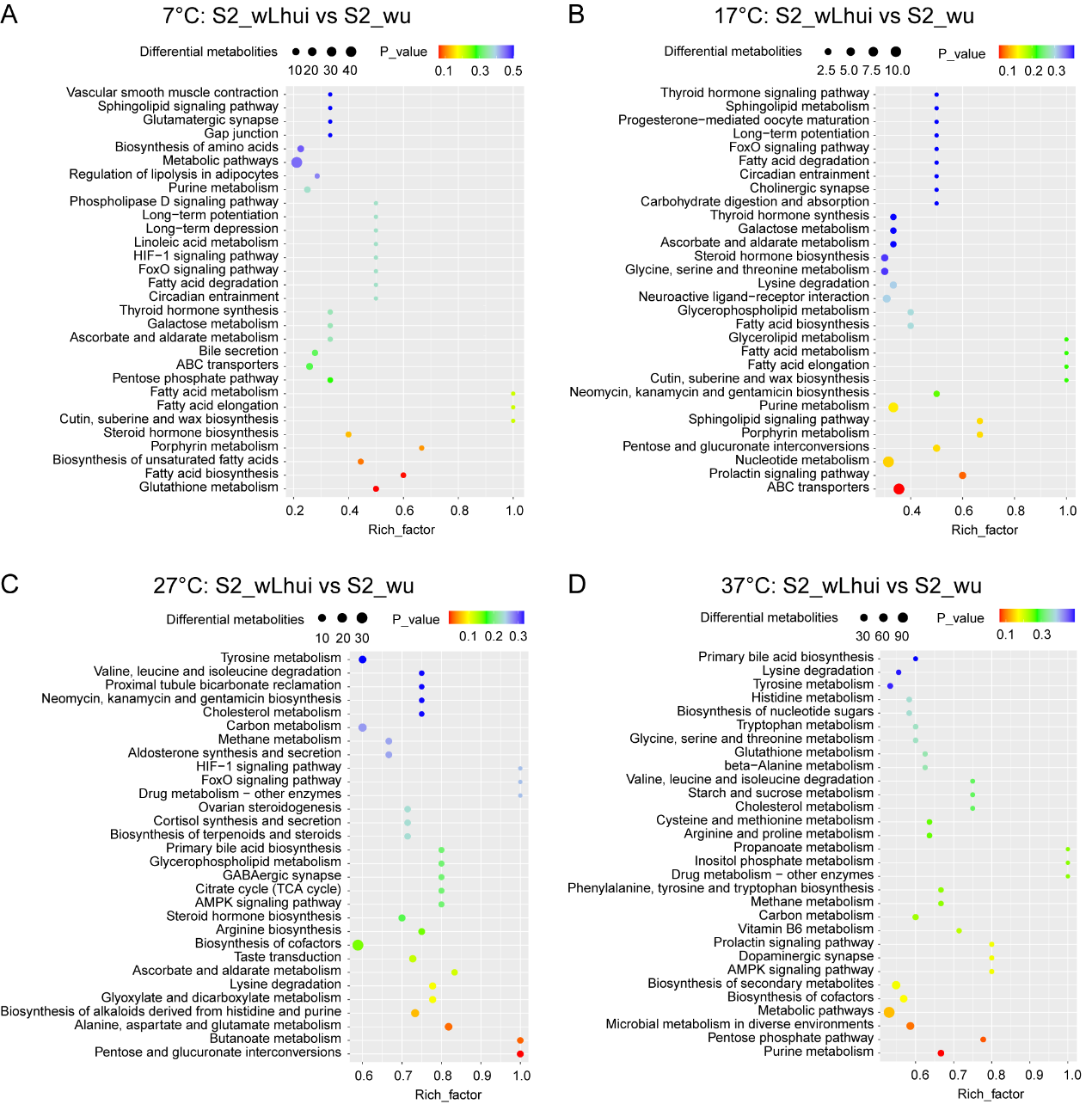


FIGURE S6 KEGG enrichment analysis of difference metabolites.
